# Supplementary material for: Responses of Nitrous Oxide Emissions and Bacterial Communities to Experimental Freeze–Thaw Cycles in Contrasting Soil Types
Source: Microorganisms. 2023 Feb 26;11(3):593. doi: 10.3390/microorganisms11030593 (PMC10054423; doi:10.3390/microorganisms11030593)
Supplement: Supplementary file 1 [file microorganisms-11-00593-s001.zip › microorganisms-1962000-supplementary.pdf]

Supplementary Information

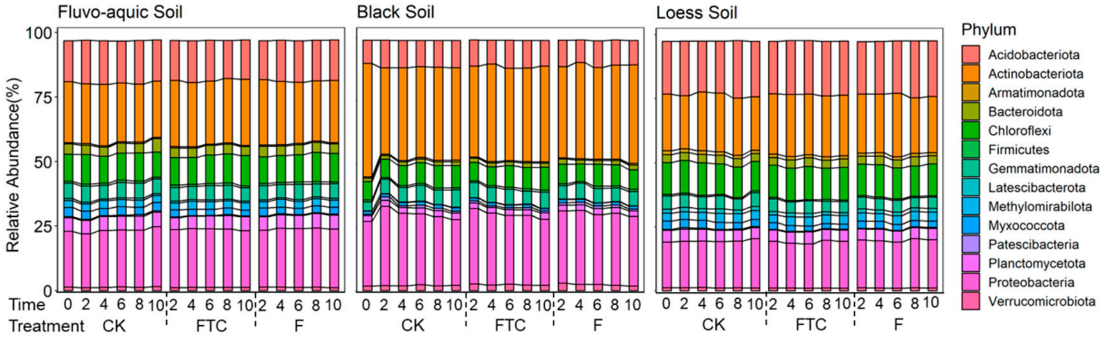

Figure S1. The relative abundances of bacterial phyla in the three soils under the FTC, F and CK treatments at different incubation times. CK: control treatment (10℃); F: freezing treatment (-10℃); FTC: freeze–thaw cycles (-10/10℃).

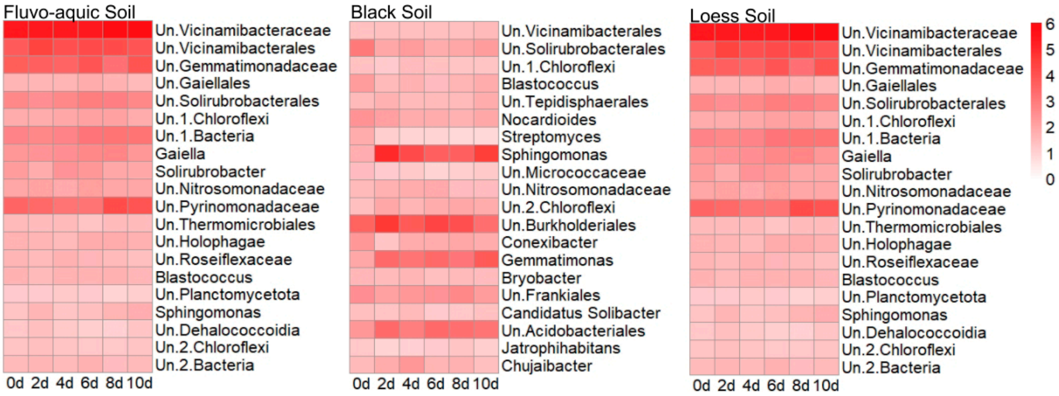

Figure S2. The top 20 dominant genera with the highest relative abundance in the three original soils (0 d) and in the CK treatment at different incubation time.

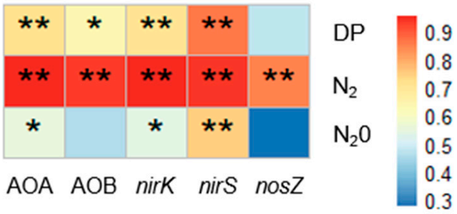

Figure S3. The Pearson correlation between nitrification and denitrification functional genes abundance of the original soil and gas production rates in the CK treatment, * 0.01 < *p* < 0.05, ** 0.001 < *p* < 0.01; AOA: AOA *amoA* gene; AOB: AOB *amoA* gene; DP: denitrification potential.
